# A Regression Approach to Model Refractive Index Measurements of Novel 3D Printable Photocurable Resins for Micro-Optofluidic Applications

**DOI:** 10.3390/polym15122690

**Published:** 2023-06-15

**Authors:** Lorena Saitta, Emanuela Cutuli, Giovanni Celano, Claudio Tosto, Giovanna Stella, Gianluca Cicala, Maide Bucolo

**Affiliations:** 1 Department of Civil Engineering and Architecture, University of Catania, Viale Andrea Doria 6, 95125 Catania, Italy; giovanni.celano@unict.it (G.C.); claudio.tosto@unict.it (C.T.); gianluca.cicala@unict.it (G.C.); 2 Department of Electrical Electronic and Computer Science Engineering, University of Catania, Viale Andrea Doria 6, 95125 Catania, Italy; emanuela.cutuli@phd.unict.it (E.C.); giovanna.stella@phd.unict.it (G.S.); maide.bucolo@unict.it (M.B.); 3 INSTM-UDR CT, Viale Andrea Doria 6, 95125 Catania, Italy

**Keywords:** refractive index measurement, photocurable resin, 3D printing, vat-photopolymerization, micro-optics, microfluidics

## Abstract

In this work, a quadratic polynomial regression model was developed to aid practitioners in the determination of the refractive index value of transparent 3D printable photocurable resins usable for micro-optofluidic applications. The model was experimentally determined by correlating empirical optical transmission measurements (the *dependent variable*) to known refractive index values (the *independent variable*) of photocurable materials used in optics, thus obtaining a related regression equation. In detail, a novel, simple, and cost-effective experimental setup is proposed in this study for the first time for collecting the transmission measurements of smooth 3D printed samples (roughness ranging between 0.04 and 2 μm). The model was further used to determine the unknown refractive index value of novel photocurable resins applicable in vat photopolymerization (VP) 3D printing techniques for manufacturing micro-optofluidic (MoF) devices. In the end, this study proved how knowledge of this parameter allowed us to compare and interpret collected empirical optical data from microfluidic devices made of more traditional materials, i.e., Poly(dimethylsiloxane) (PDMS), up to novel 3D printable photocurable resins suitable for biological and biomedical applications. Thus, the developed model also provides a quick method to evaluate the suitability of novel 3D printable resins for MoF device fabrication within a well-defined range of refractive index values (1.56; 1.70).

## 1. Introduction

Since the 1990s, lab-on-a-chip (LOC) has gained great importance [1]. Indeed, by means of this type of device, characterized by the presence of micro-metric channels with disparate geometries, it is possible to analyze and test very small quantities of biological or chemical fluids. As a result, several operations that usually require conventional biochemistry laboratories, such as sample preparation for reactions and biopsies of biological fluids, can be performed [1,2]. Micro-optofluidic (MoF) devices fall within this class of devices, and they are also adopted to optically detect and control two-phase flow flowing within micro-metric channels in several fields of application, such as biomedical and chemical ones [3,4,5,6,7]. The optical approach is the most preferred since it is slightly invasive and allows for many types of measurements [8].

However, it is worthy of attention that when MoF must be used exploiting the optical approach to analyze certain fluids [9,10,11], they are characterized by a layout where the presence of optical components is properly integrated with microfluidic channels. Thus, care must be taken with the material choice during the design procedure. Indeed, for carrying out this type of analysis performed using MoF devices, it is crucial to confine and transport the beam light coming from a light source towards the small volume of fluid circulating within micro-channels. In the end, properly acquiring the beam of light passing through the fluid makes it possible to collect useful non-specific information, such as its density, viscosity, velocity, and so on [12]. Therefore, to accomplish this purpose, the materials used for manufacturing this class of devices must have excellent optical properties to avoid issues such as high losses. The gold standard material traditionally used for fabricating optical components was glass to fulfill these needs. In the past, processes of etching, deposition, photolithography, and so on were followed to manufacture microfluidic devices in quarts, silicon, and, once again, glass [13,14,15,16,17]. However, new technical strategies were needed because of many experienced drawbacks, such as limited geometries realization, expensive manufacturing processes due to the need to use cleanrooms and high costs for raw materials supply. For this reason, all the aforementioned demands and drawbacks related to more traditional manufacturing processes and raw materials were overcome by a new trend that relies on using polymer materials for 3D printing. Indeed, this strategy allowed the realization of cheaper MoF devices, characterized by complex geometric models that are difficult or impossible to produce by more conventional manufacturing technologies, suitable for mass production. Among the existing 3D printing techniques, which have ensured enhancements both in terms of resolutions and capabilities for microfabrication [18,19,20,21,22], stereolithography (SLA), MultiJet Printing (MJP), and fused deposition modeling (FDM) are the most used ones. Among the latter techniques, the SLA is the most performing one since it permits higher resolution, tighter tolerances, and compatibility with thermoset polymers, which are classified as optical-grade polymers [23] compared to MJP and FDM. For example, *Saitta* et al. [10] previously realized a MoF device for slug-flow detection in one piece by using a one-step manufacturing approach and achieving down-to-micron precision as an alternative to stereolithography. Moreover, the first 3D printed optical components made of plastic, dating back to 2015, were initially realized using thermoplastic materials, such as acrylonitrile butadiene styrene (ABS) and polyethylene terephthalate glycol (PETG). These materials were chosen for their commoditization and easy accessibility. However, their optical performance was found to be very limited due to significant losses in optical quality. This is why novel resins classified as optical-grade polymers (i.e., affected by low optical losses) were introduced [24]. The latter are cyclic olefin polymers (COP), poly(methylmethacrylate) (PMMA), polycarbonate (PC), and poly(styrene) (PS). In the last decade, Poly(dimethylsiloxane) (PDMS) has been selected as the preferred choice [25] due to its suitability for optical detection, which is related to its transparency and quite low refractive index value, i.e., *n* = 1.4. However, this polymer is suitable for casting fabrication techniques, such as soft lithography [26], for realizing micro and nano-structures. But, this type of manufacturing technique does not allow for the realization of devices in one piece, thus requiring the assembly of complex structures, causing issues such as poor bonding, imperfect alignment, etc. In this sense, the 3D printing approach may help overcome the aforementioned drawbacks by providing the opportunity to directly realize 3D objects by exploiting a layer-by-layer approach. For this reason, *Fleck* et al. [27] developed a high-resolution PDMS resin using a methacrylate–PDMS copolymer and the novel combination of a photoabsorber called Sudan I and a photosensitizer known as 2-Isopropylthioxanthone. This formulation exhibited a transmission >75%, which was combined with a digital light processing (DLP) 3D printer to assess the possibility of fabricating truly micron-scale parts with channels as small as 60 μm tall. Other examples of novel 3D printable polymer materials relying on photopolymerization processes have been developed by *Zhu* et al. [28] and *Shi* et al. [29].

Furthermore, there is a need for the development of novel transparent resin formulations that exhibit good optical properties and are compatible with vat photopolymerization (VP) 3D printing techniques. These advancements would enable the manufacturing of micro-parts with high accuracy and precision. The selection of materials with good optical properties, such as high transparency, high transmission percentage, and low refractive index, is essential in LOC to implement optical detection methods for analyzing biological or chemical samples [30,31,32,33,34].

Generally, the VP 3D printing techniques represent optimal manufacturing processes for various use cases across a wide variety of biotechnology industries, such as medical devices [35,36], drug delivery systems [37,38], and microfluidic devices [39,40]. The field of 3D printing offers numerous advantages that contribute to its attractiveness, including high print resolution, relatively high 3D printing speed, fairly low production cost, smooth 3D printed surfaces, and very high accuracy and fabrication precision, allowing for more intricate, exact, and replicable parts [38,41,42,43]. Among the VP 3D printing methods, digital light processing (DLP) and stereolithography (SLA) have shown an increasing potential for applications in the biotechnology field and, more specifically, in the LOC field due to the aforementioned benefits that distinguish them. DLP is a 3D printing technique whereby the photocurable resin is cured by a projector, which is the light source that projects an image onto the resin by curing the entire projected shape at once. Thus, in this case, the resolution, accuracy, and precision are determined by the projector’s resolution (the current standard is 1080P), the optical character of the projection lens, and the tightness of the mechanical XYZ movement [44,45]. In the end, this 3D printing technique generally exploits a bottom-up approach. Hence, the use of support is required to secure the part of the building platform. Conversely, the SLA process exploits a top–down approach where it is not required to use supports in order to secure the bottom surface to the print surface. Furthermore, unlike the DLP 3D printing process, SLA processes use a laser to deliver concentrated light into a vat of resin, thus photopolymerizing the latter to fabricate a solid layer of the final 3D printed object in accordance with the initial design. Hence, XY resolution combines the laser’s spot size and the increments by which the laser beam can be controlled [46].

In addition to these, among the VP techniques, an innovative technique has been recently patented (US Patent 9492969), which is named Projection Micro Stereolithography (PμSTL). It has been developed as a hybrid technique combining the advantages of DLP and SLA 3D printing processes to develop a 3D printing technique characterized by a higher level of resolution while simultaneously keeping unchanged the capability to manufacture large parts with a high level of tolerance and ultra-high resolution (down to 10 μm) [47].

Within the LOC field of application, for realizing MoF devices integrating optical elements, it becomes very crucial to determine how the novel 3D printable materials interact with electromagnetic waves. Accessing a knowledge base of optical properties is paramount, even though this information is not generally provided in the technical datasheet (TDS) of novel commercialized resins. In detail, it is important to run quantitative measures of how a material interacts with light. Thus, large efforts must be made to develop simple methodologies to estimate certain types of optical properties of interest, such as the refractive index value. Knowing the accurate value of the latter parameter of a material is crucial to make some predictions (thanks to simulation tools), allowing the prediction of the photon interaction, which may be reflection, absorption, or scattering at the interface [48]. In detail, the refractive index is a measure of the bending of light when moving from one medium into another. It is also defined as the ratio of the velocity of light in a vacuum to the velocity of light in a medium [49].

Many studies have been run over the years to provide refractive index value determination methods to meet this need [50,51,52,53,54,55,56,57]. However, most of them are suitable for analyzing biological or liquid samples. In accordance with the current state-of-the-art, accurate characterization methods such as refractometry, ellipsometry, and prism coupling measurements are commonly employed to assess the optical properties of samples. However, these methods are often expensive and highly demanding regarding the macro- and micro-scale planarity (very smooth surfaces) of the samples [58,59,60,61]. To solve this problem, *R. J. Nussbaumer* et al. [62] have proposed an immersion method to determine the refractive index of rough solids. This method relies on simple transmission measurements using an ordinary UV/vis spectrometer, avoiding the complex approach of searching for different liquids matching the solid’s refractive index [57,63,64].

Starting from the latter work, the novelty of this experimental study consists of developing a novel polynomial regression model to enable refractive index measurements suitable for transparent 3D printable resins. In addition, to the best of our knowledge, a novel, simple, cost-effective, and efficient experimental setup is presented in this study for the first time. The proposed novel approach relies on ordinary transmission measurements and avoids sample immersion within a liquid to determine the considered optical property. In fact, the transmission measurements were conducted with air as the surrounding medium. This decision was justified by the fact that VA photopolymerization yields a high-quality surface finish characterized by a quite low roughness ranging between 0.04 and 2 μm. Furthermore, even the selected materials to develop the polynomial regression model, optical photocurable adhesives with a known and certified refractive index, presented a very low surface roughness (nanometric scale) [51] once the cross-linking process was completed.

In this work, a *quadratic polynomial regression model* has been developed to correlate the known *refractive index value (R)* of fully cured photocurable adhesives (NOA resins), chosen as an independent variable or predictor, to their empirically measured *transmission value (T)*, selected as a dependent variable or response. As soon as the regression model was empirically developed for a well-defined operative range for the refractive index parameter, i.e., ranging between 1.56 and 1.70, its equation was inverted. This inversion determined the unknown refractive index value of photocurable 3D printable resins, starting from their measured transmission value.

In the end, the optical performances of two different MoF devices were examined as an application in the LOC field. These devices were constructed using novel 3D printable resins and fabricated using an innovative VA 3D printing technique called Projection Micro Stereolithography (PμSTL). The optical performances of the 3D printed MoF devices were evaluated alongside those of a PDMS-based MoF device to provide a basis for comparison. The latter decision is justified by the fact that PDMS is considered the gold standard polymer material for both optical and microfluidic applications.

The obtained results allowed for the identification of a well-defined range of refractive index values for novel resins developed for LOC applications. Additionally, a simple and effective equation was derived to estimate their unknown refractive index value from empirical transmission measurements.

## 2. Materials and Methods

### 2.1. Materials

To experimentally determine the regression model that correlates the empirically measured transmission value of full-cured photocurable resins with their refractive index values, four different optical photocurable resins with a known refractive index value were selected:

(i). *NOA88*—it is a low-viscosity (250 cps) UV-curing adhesive with a refractive index equal to 1.56 at 589 nm, requiring 5 J/cm^2^ of energy to fully cure a 25–50 micron bond, and having an absorption range ranging between 315 and 395 nm. According to the provided SDS (safety data sheet), it is a proprietary urethane-related resin-based formulation composed of a mixture of mercapto esters (the content of 50–65%) and triallyl isocyanuarte (the content of 30–55%);
(ii). *NOA160*—it is a high-viscosity (2200 cps) UV-curing adhesive with a refractive index equal to 1.60 at 589 nm, requiring 6 J/cm^2^ of energy to fully cure a 25–50-micron bond. In accordance with the provided SDS (safety data sheet), it is a proprietary urethane-related resin-based formulation composed of a mixture of pentaerythritol tetra 3 (the content of 30–50%) and acrylic monomer (the content of 30–60%);
(iii). *NOA1665*—it is a UV-curing adhesive characterized by a viscosity of 800–1000 cps, with a refractive index equal to 1.665 at 589 nm, which requires 6 J/cm^2^ of energy to fully cure a 25–50-micron bond. In line with the information provided by the SDS (safety data sheet), it is a proprietary urethane-related resin-based formulation composed of a mixture of metal oxide (the content of 30–60%) and acrylated resin (the content of 35–45%);
(iv). *NOA170*—it is a very high-viscosity (4500–5500 cps) UV-curing adhesive characterized by a high refractive index, i.e., equal to 1.70 at 589 nm, that needs 6 J/cm^2^ of energy to fully cure a 25–50-micron bond. Based on the supplied SDS (safety data sheet), it is a proprietary acrylated-related resin-based formulation composed of a mixture of acrylated resin (content of 40–60%) and acrylic monomer (content of 10–30%).

The four selected Norland Optical Adhesives (NOA) for the study were purchased from Edmund Optics LTD (Nether Poppleton, York, UK), and their properties are summarized in Table 1.

Moreover, by using a casting procedure into a silicon mold, the upper surface (exposed to the air) of the NOA88 fabricated samples was characterized by a surface roughness expressed as average roughness (Ra), root mean square roughness (RMS), and peak-to-peak equal to 12.33 nm, 7.18 nm, and 140.12 nm, respectively. The authors previously determined the latter using atomic force microscopy (AFM) [51]. Similar results for the roughness can be assumed for the other NOA resin specimens since they were manufactured using the same procedure.

Next, three different materials were chosen to manufacture the micro-optofluidic devices for two-phase flow detection. The first device (called *Device 1* henceforth) was fabricated using the SYLGARDTM 184 Silicone Elastomer Kit. The latter presents a chemical composition that is a mixture of both a Poly(dimethylsiloxane) (PDMS) elastomer and a suitable curing agent and is also characterized by a refractive index value of 1.4118 at 589 nm (as declared by the provided TDS). It was purchased from Farnell Italia S.R.L. (Sylgard 184 elastomer kit, Dow Corning, 148 Midland, MI, USA). Moving on, both the second (called *Device 2* from now on) and the third (called *Device 3* henceforward) devices were realized by exploiting the highly innovative micro-precision 3D printing technique named Projection Micro Stereolithography (PμSL) and commercialized by *Boston Micro Fabrication Materials Technology Co., Ltd.* (BMF, Maynard, MA, USA)*. Device 2* and *Device 3* were made of photocurable resins named HTL resin and BIO resin, respectively. The first one (HTL) is a high-performance, rigid, and heat-resistant yellow resin proprietary developed by Boston Micro Fabrication (BMF, Maynard, MA, USA).

Moreover, this resin grade is characterized by good temperature stability and mechanical properties, which make it suitable for autoclave sterilization. Next, in agreement with its TDS, this resin shows a heat distortion temperature (HDT) of 140.7 °C (@0.45 MPa) and a glass transition temperature (Tg) of 172 °C. The second photocurable resin (BIO) is a biocompatible resin suitable for non-implantable medical applications and can also undergo sterilization. It has passed several ISO 10993 biocompatibility tests (such as skin irritation and sensitization, toxicity, cytotoxicity, pyrogenicity, and in vitro hemolysis). It has shown a cell culture survival rate in vitro of 75%. The latter-mentioned properties justified the reason it was selected as a raw material for micro-optofluidic devices that find application in health and biomedical engineering. Furthermore, according to its TDS, it is a yellow resin showing a HDT of 85.7 °C (@0.45 MPa), which was developed by Boston Micro Fabrication (BMF, Maynard, Massachusetts) as well.

It must be highlighted that the refractive index of the used PDMS is provided by its TDS. This is because PDMS is a widely used material for optical applications in the state-of-the-art. It is unknown for the two yellow-grade photocurable resins used via PμSL 3D printing.

In the end, the roughness for *Device 1* (PDMS) and *Device 2* (HTL resin) was already determined by the authors using the AFM analysis [10]. The previously collected results are summarized in Table 2. Since *Device 2* (HTL resin) and *Device 3* (BIO resin) were realized using the same 3D printing technique, the latter can be assumed to have the same roughness range values measured for the HTL resin, as declared by the company providing the machine.

#### Specimens Preparation

**Figure 1:**
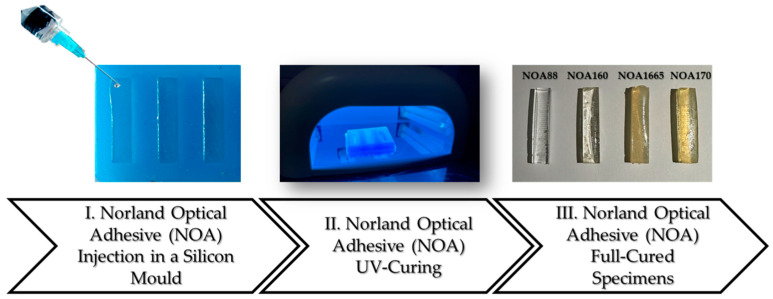
Followed the workflow for the fabrication of the used specimens for transmission measurements.

Specimens having a size equal to (10 × 6 × 4) mm^3^ were used for the transmission measurements. Using all the selected NOA materials (see Table 1), the specimens were prepared using the procedure reported in Figure 1. At first, each liquid selected for NOA was micro-injected using a syringe equipped with a 25 gauge needle (step I) within a silicone mold. Secondly, each photopolymer adhesive was irradiated with a universal lamp bulb with UVA emission at 365 nm (step II). In the end, four full-cured bars were obtained by providing the required UV-curing energy (step III).

**Figure 2:**
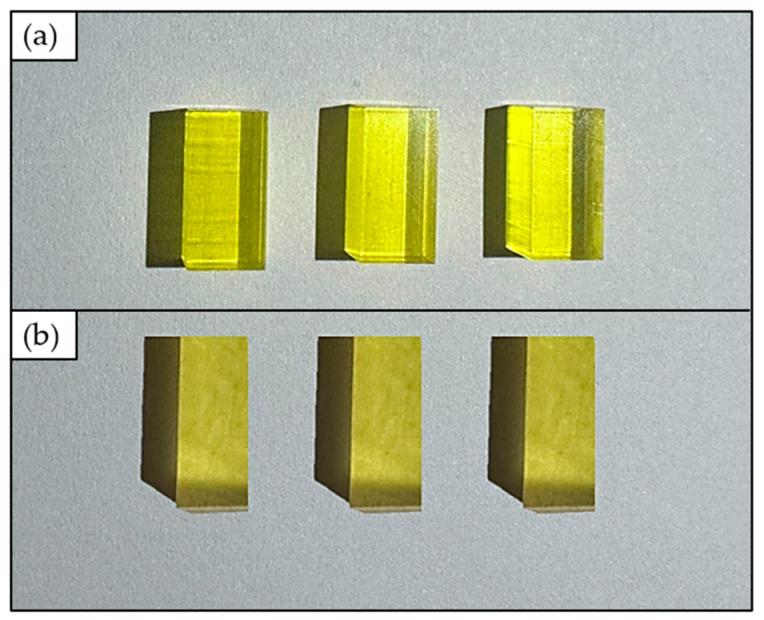
3D printed BIO (**a**) and HTL (**b**) specimens for transmission value measurements.

Next, specimens 4 mm thick made of the two yellow-grade photocurable 3D printable resins (BIO and HTL resins) were 3D printed using a microArch^®^S140 ultra-high resolution (10 μm) 3D printer (BMF, Maynard, MA, USA) for transmission measurements. The final 3D printed specimens are shown in Figure 2.

### 2.2. Transmission Measurements: Experimental Setup

**Figure 3:**
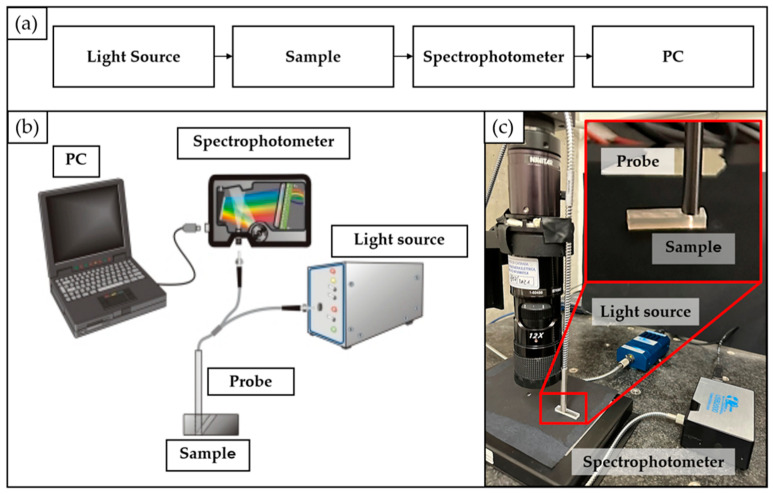
Experimental setup for transmission measurements: (**a**) block scheme; (**b**) schematic representation; (**c**) real picture.

A block diagram and a schematic representation of the used transmission measurement system are shown in Figure 3a and Figure 3b, respectively. In particular, it is composed of (i) the source light; (ii) the probe; (iii) the material sample; (iv) the spectrophotometer; and (v) a PC for the measurement acquisition through dedicated software.

The actual experimental setup is shown in Figure 3c. Specifically, a halogen light source (LS-1 Tungsten Halogen Light Source, Ocean Optics, Dunedin, FL, USA), providing visible light, was connected through a SMA connector to the probe (Avantes, Lafayette, CO, USA). The light beam coming from the light source was sent through an illumination fiber to the sample, and the reflection was measured by using a second fiber in the center of the probe’s tip. The latter was positioned in close contact with the sample’s surface (as shown in Figure 3c insert) to prevent the existence of different materials between the probe and the sample itself. This setup was implemented to minimize any potential impact on the transmission measurement that could arise from the presence of different materials. The second fiber was coupled through a SMA connector to a spectrophotometer (USB2000, Ocean Optics, Dunedin, FL, USA), configured to the appropriate wavelength range of interest (i.e., 200–1100 nm). The spectrophotometer was connected via a USB cable to the PC to collect the transmission measurements through the Spectra Suite dedicated software 2.0.

*Transmission* is defined as the percentage of energy passing through a system relative to the amount that passes through a reference sample. In the measurement protocol, to calibrate the instrument, the air was selected as a reference sample with a thickness equal to the investigated material samples (4 mm). The latter was associated with a transmission value of 100%. After the calibration phase, the transmission measurements were performed and acquired statically for each selected material sample (see Table 1), following the replicated general factorial design fully described in Section 2.3.

The collected transmission measurements were exported as a *.ProcSpec* file and then processed in MATLAB (MathWorks^®^). Specifically, the “*spectrum. wavelength*” and the “*spectrum.processedPixels*” MATLAB (MathWorks^®^) struct fields, corresponding to the wavelength and the transmission (in percentage), respectively, were considered to track the transmission measurement signal.

The transmission values used for the regression model development were extracted in correspondence with a wavelength equal to 589 nm to directly compare with the known refractive index values of the examined NOA resins. Indeed, according to their TDS, they are in the same condition.

### 2.3. Regression Model for Refractive Index Estimation

In this experimental study, the functional relationship existing between the known *refractive index value* (*R*) of fully cured photocurable adhesives (NOA resins), chosen as *independent variables* or *predictors*, and their empirically measured *transmission value (T)*, selected as a *dependent variable* or *response*, was estimated. A *quadratic polynomial regression model* has been considered:

*T_i_* = β_0_ + β_1_ *R_i_* + β_11_ *R_i_*^2^, (1)

where β_0,_ β_1_ and β_11_ are the *regression coefficients*. The matrix notation corresponding to this model is:

***T*** = ***R* β**, (2)

where ***T*** is the (44 × 1) vector of observations, i.e., the measured transmission value; ***R*** is the (44 × 3) design matrix; and **β** is the (3 × 1) vector of the regression coefficients.

For the test procedure, only one design factor (independent variable) was considered, i.e., the type of material (factor A), which was varied among four different levels (*a* = 4). The *a* = 4 levels correspond to NOA88, NOA160, NOA1665, and NOA170. The measured transmission value for each selected material was considered as the response variable. The number of replications was equal to *n* = 11, for a total of *N* = *a · n* = 44 runs. The experimental plan is summarized in Table 3.

An analysis of variance (ANOVA) study was run to validate model assumptions in regression.

After developing the empirical regression model for a well-defined operative range for the refractive index parameter, i.e., ranging between 1.56 and 1.70, Equation (1) was properly inverted. This inversion process resulted in the derivation of an empirical equation to predict the unknown *refractive index value* for the photocurable 3D printable resins, starting from their measured *transmission value*:

β_11_ *R*^2^ + β_1_ *R* + β_0_ − *T* = 0, (3)

The roots of the second-order equation have been determined by exploiting the *roots* function in MATLAB (MathWorks^®^), which allows calculating the roots of a single-variable polynomial represented by a vector of coefficients. In detail, three acquisitions for the measured transmission value *T* were run for the two selected photocurable 3D printable resins (i.e., HTL and BIO). Using Equation (3), the estimated refractive index value *R* was determined for each acquisition. Finally, its estimate was expressed as its mean ± st. deviation.

### 2.4. Application: Micro-Optofluidic Device for Slug-Flow Detection

Once the estimated refractive index value was determined for the two novel photocurable 3D printable resins (BIO and HTL resins), this information was used to assess their suitability for micro-optofluidic device fabrication. For this purpose, a micro-optofluidic (MoF) device for two-phase fluid detection, which had been previously designed by the authors in earlier works [9,10,11], was manufactured through a direct 3D printing process using the two different investigated resins.

Two-phase flow refers to two immiscible fluids, such as gas–liquid, immiscible liquid–liquid, or liquid and microparticles, one dispersed in the other and circulating into the same micro-system [8,65]. Within this context, the optical method is worthy of consideration since it is very strategic for carrying out many less invasive measurements. The MoF device’s working principle relies on the absorption phenomenon. This means that when the incident laser beam interacts with the two different phases present in the device, which are characterized by two different refractive index values, the transmission of light exhibits different characteristics. The nature of light transmission strongly depends on the fluid with which it interacts at a precise instant.

The optical properties of the two yellow-grade novel photocurable resins (BIO and HTL), related to their estimated refractive index values, were then compared with the PDMS ones. It is justified by the fact that the latter is the most commonly used polymeric material for optical applications since its refractive index is close to the glass one (1.4118 for PDMS versus 1.52 for glass), which is considered the gold standard material.

**Figure 4:**
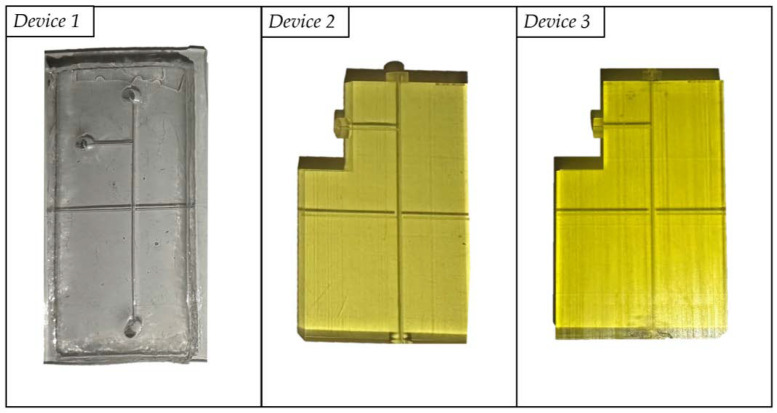
Manufactured micro-optofluidic devices made of PDMS (*Device 1*), HTL (*Device 2*), and BIO (*Device 3*) resins.

In detail, three different devices were fabricated, with variations in the manufacturing technique and the material used for their construction. Their optical performances were compared and tested according to the procedure described in Section 2.4.1. under the same operative conditions. *Device 1*, made of PDMS, was fabricated through an ad hoc master-slave fabrication approach relying on 3D printing, as previously explained elsewhere by the authors [1,2,3]. Both *Device 2* and *Device 3* were realized by exploiting the highly innovative micro-precision 3D printing technique, PμSL. The three fabricated devices are shown in Figure 4.

The detailed workflow followed for the three devices’ manufacturing has already been explained elsewhere by the authors [9,10].

#### 2.4.1. Optical Detection: Experimental Setup and Signals Processing

**Figure 5:**
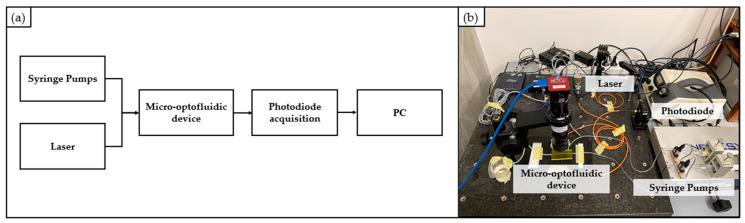
Experimental Setup for Optical Measurements: (**a**) block scheme; (**b**) real picture.

To assess the optical properties of the investigated photocurable 3D printable resins selected for the realization of the MoF devices, the optical signals were acquired by pumping either deionized water (characterized by a refractive index *n*_Water_ = 1.3) or air (characterized by a refractive index *n*_Air_ = 1.0), into the micro-channel. The micro-channels were composed of three different materials: PDMS (*Device 1*), HTL resin (*Device 2*), and BIO resin (*Device 3*). Both air and water were injected within the micro-optofluidic device’s micro-channel through a syringe pump system (neMESYS low-pressure module, Cetoni, GmbH, Korbussen, Germany). A laser system (NovaPro 660–125, RGB Lasersystems, Kelhein, Germany) with an emission wavelength of 660 nm was used as a light source to detect the static fluid inside the micro-channel by exploiting three levels of input power, i.e., 1 mW, 3 mW, and 5 mW. The micro-optofluidic device was coupled with two 365 μm diameter optical fibers. The former was connected to the laser system for optical actuation. While, the latter, which was connected to a photodiode (PDA100A, Thorlabs, Newton, NJ, USA) with a gain of 40 dB, was used for the detection,. A PC oscilloscope (Picoscope 2204A, Pico Technology, Cambridgeshire, UK) with a sampling frequency of 1.5 kHz was used to acquire the detected signals. Figure 5 reports the blocking scheme and the real picture of the experimental setup for detecting optical signals.

For each investigated scenario, the optical signal was acquired for a period of 30 s. Next, the acquired signals were post-processed in MATLAB (MathWorks^®^). In particular, a low-pass filter with a 40 Hz cut-off frequency was applied to eliminate high-frequency components. Then, a smoothing procedure was applied to remove the noise from the signal and reveal the main pattern. In this way, a single sample of V(i) observations was obtained for each voltage-acquired signal for *i* = 1, …, *N_v,_* and *N_v_* = 45,000. After this, the corresponding sample mean values (${\bar{V}}_{Water}$ and ${\bar{V}}_{Air}$) and the sample standard deviations were evaluated for each sample V(i). In the end, the voltage range (ΔV), defined as the difference between the average of the water signal (${\bar{V}}_{Water}$) and the average of the air signal (${\bar{V}}_{Air}$), was evaluated to prove that the optical part of the device is really able to discriminate between two different fluids (air and water). In fact, due to the difference between their refractive index values (1.3 for water versus 1.0 for air), the signals were detected at two different voltage values using the same laser input power: a higher voltage value for water and a lower voltage value for air.

## 3. Results and Discussion

### 3.1. Transmission Measurements Results

**Figure 6:**
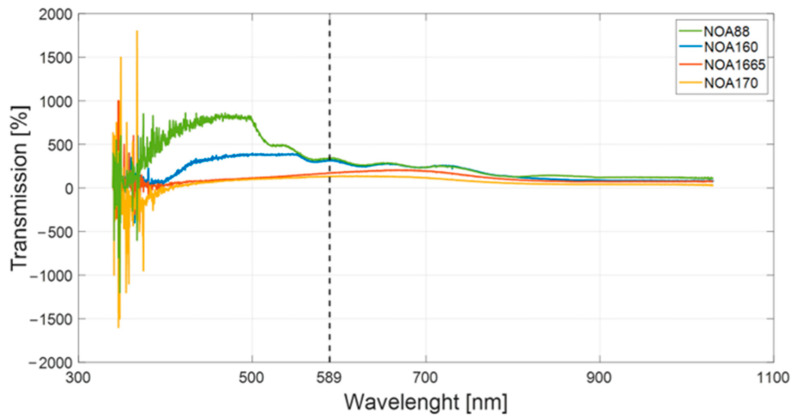
Average acquired transmission spectra for the analyzed Norland Optical Adhesives (NOA) adhesives.

The average acquired transmission spectra for the four investigated materials are reported in Figure 6 in correspondence with the whole wavelength range of interest (i.e., 200–1100 nm).

The transmission value at a wavelength of 589 nm was extracted for each acquired spectrum, as reported in Figure 6 with the vertically dashed black line.

**Figure 7:**
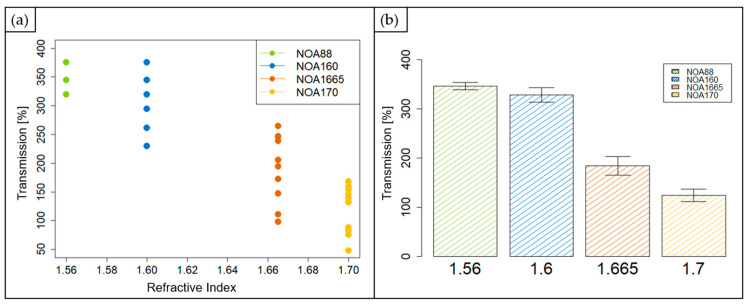
Scatter plot of the collected transmission measurements at 589 nm (**a**); bars plot showing the average transmission value for the four materials. Bar errors represent the standard errors of the collected measurements (**b**).

The collected measures for each investigated material are reported in the scatter plot in Figure 7a. The calculated average values and standard errors for the photocurable NOA resins are summarized in the bar plot shown in Figure 7b. According to the results, the NOA88 (in green in Figure 7) showed the highest average transmission value, 346.68 ± 7.44. The NOA160 (in blue in Figure 7) presented a decreased value for the transmission (328.57 ± 15.01) of about 5%. Next, for the NOA1665 (in orange in Figure 7), an average transmission value of 183.76 ± 28.93 was measured, reduced by about 44% and 47% compared to the NOA88 and the NOA160, respectively. In the end, the lowest average transmission value was recorded for the NOA170 (in yellow in Figure 7), equal to 122.65 ± 12.75, thus having a lower value of about 65% than the NOA88. The obtained decreasing trend is justified by the photocurable NOAs’ refractive index values. The higher the refractive index value of the investigated material, the lower the measured transmission value. Indeed, starting from NOA88, which is fully transparent, the other considered resins became more matt and yellow-like.

Furthermore, it must be highlighted that a lower standard error affects the measured transmission value of the NOA88 material sample. This is due to its greater homogeneity and full transparency, as shown in Figure 1.

### 3.2. Quadratic Polynomial Regression Model for Refractive Index Value Determination

The regression equation unveiling the true functional relationship existing between the known *refractive index value* (*R*) and the measured *transmission value* (*T*) of the fully-cured photocurable adhesives (NOA resins) is equal to:

*T* = −15,382 + 20,903 *R* − 6933 *R*^2^, (4)

**Figure 8:**
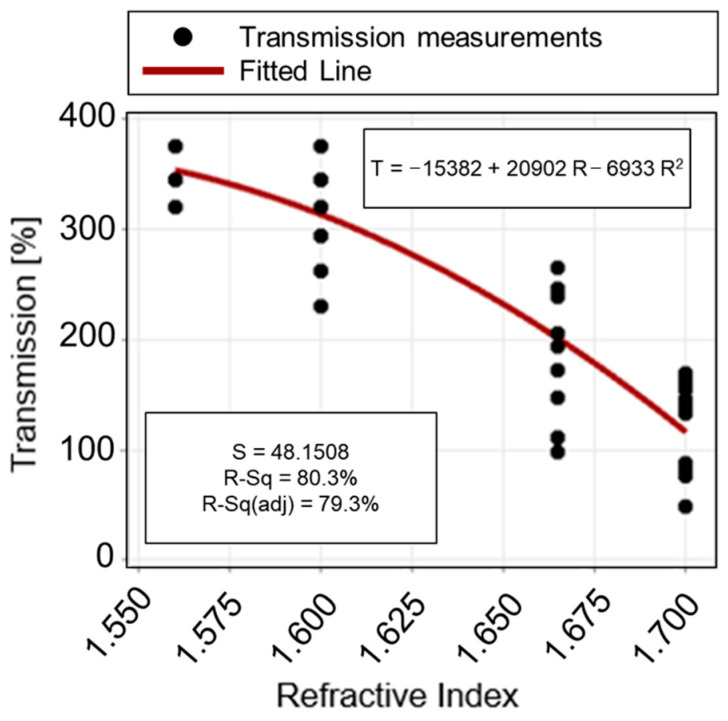
Fitted line plot: selected quadratic polynomial regression model fitting the collected observations (transmission values for NOA materials). *R = Refractive index* value of the *predictor,* and *T = Transmission* value of the investigated *response*.

**Figure 9:**
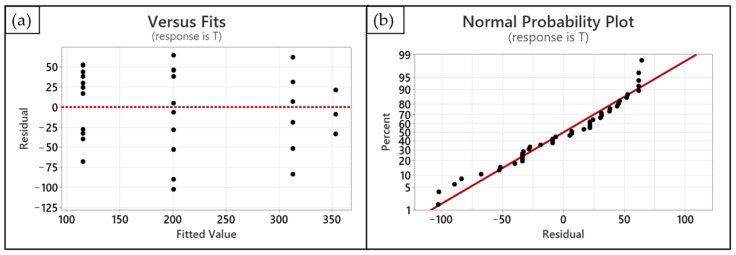
Residual analysis: residual versus fitted value plot (**a**) and normal probability plot for residual values (**b**).

An ANOVA study was performed to investigate the statistical significance of each term, i.e., both the linear and quadratic ones. The regression model is significant from the ANOVA study shown in Table 4 (*p*-value < 0.05). Moreover, since the values for *R*^2^ (80.28%) and *R*^2^-adj (79.31%) are quite high, it is possible to assume that the model is adequate since the noise level is very low. As a result, the model fits well with the collected observations. In accordance with the ANOVA results (Table 4), it is possible to notice that the linear term of the model is absolutely significant (*p*-value < 0.05). On the other hand, the quadratic term is slightly less significant (*p*-value = 0.074 > 0.05). However, it was added to the model since it allows a better fitting of the observations related to the lowest value (*R* = 1.56) of the operative range selected for the refractive index parameter. Figure 8 shows the fitting quadratic regression line. Figure 9 shows the model adequacy check by plotting residuals from the regression model versus fitted values and their normal probability plot. The two plots reveal no anomaly.

Next, by inverting the regression model Equation (4), an expression is derived to predict the unknown *refractive index value* for the photocurable 3D printable resins, starting from their measured *transmission value*:

6933 *R*^2^ − 20,902 *R* + (15,382 + *T*) = 0, (5)

Three observations of the *transmission value* were collected for the BIO and HTL resins, respectively. The collected observations for the two considered photocurable 3D printable resins are reported in Table 5. Next, the roots of Equation (5) were calculated to determine the estimated refractive index value ($\hat{R}$) for each replication. In detail, two roots were determined for each considered resin; their values are reported in Table 6. The first root (${\hat{R}}_{1}$) was never considered since it is broadly lower than the inferior limit of the operative range for the refractive index value (equal to 1.56). Conversely, the obtained values for ${\hat{R}}_{2}$ are strictly close to the upper limit (1.70). Moreover, since the investigated resins BIO and HTL are yellow-like (see Figure 2), as well as the NOA170 resin (see Figure 1), it is reasonable to consider the root ${\hat{R}}_{2}$, which is close in value to the refractive index of NOA170 resin.

**Figure 10:**
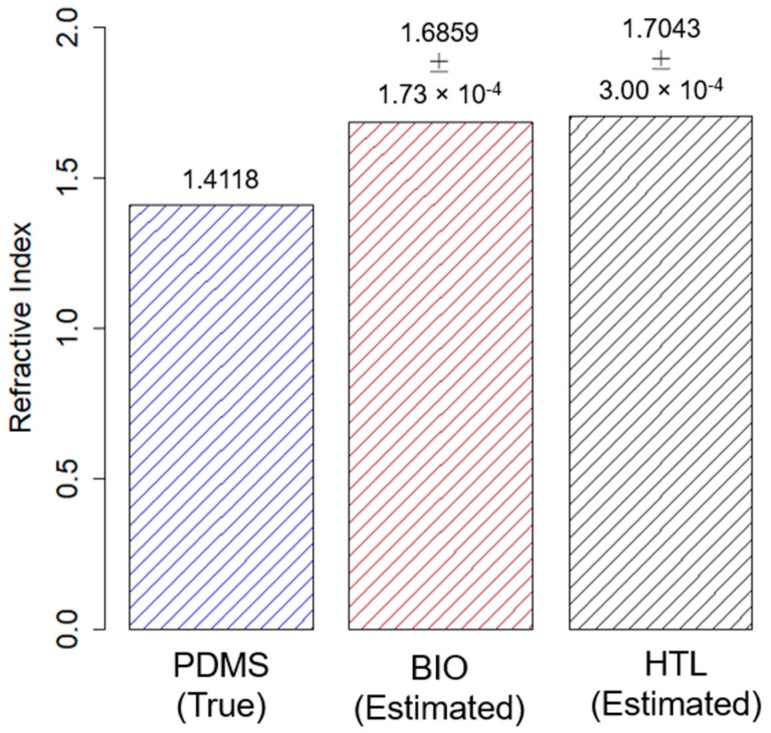
Bars plot for the estimated mean value of the refractive index parameter for photocurable 3D printable resins BIO and HTL, compared to the true value from the technical data sheet (TDS) for the PDMS. Bar errors were not added because they were significantly narrower than the bar height.

The means of estimated refractive index values for the 3D printable BIO and HTL resins are shown in Figure 10, where they are also compared with the true value for the refractive index of PDMS (provided by its TDS).

### 3.3. MoF Devices Optical Signals

**Figure 11:**
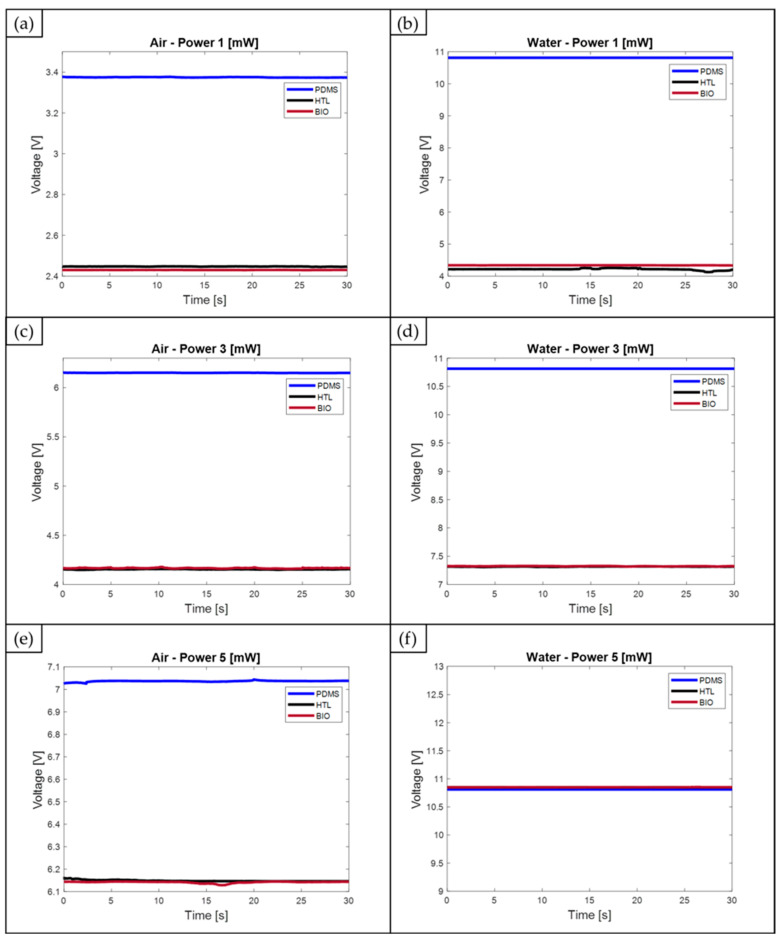
Acquired optical signals by means of the three MoF devices made of PDMS, BIO, and HTL resins: air detection at a laser power of 1 mW (**a**), 3 mW (**c**), and 5 mW (**e**); water detection at a laser power of 1 mW (**b**), 3 mW (**d**), and 5 mW (**f**).

The acquired optical signals by varying the fluid—air and water—injected within the MoF device’s channels and the selected input laser power, 1, 3, and 5 mW, are reported in Figure 11a–f. Based on the obtained results, it was assessed that the estimated refractive index values for the two novel photocurable and 3D printable resins, BIO (*n*_BIO_ = 1.6859) and HTL (*n*_HTL_ = 1.7043), are higher than those of PDMS by approximately 19% and 21%, respectively. However, despite this difference, it was concluded that both resins are still suitable for the intended purpose. Using these two polymer resins for manufacturing the MoF devices for two-phase flow detection, it was still possible to acquire valuable optical signals while detecting both air and water in correspondence with the three levels of laser input power, according to the sample mean values reported in Table 7 for each investigated scenario.

As shown in Figure 11a, the air voltage value at an input laser power of 1 mW is about 28% lower for the MoF devices made of BIO and HTL resins compared to the one detected from the PDMS device under the same conditions. By increasing the laser power to a value of 3 mW (Figure 11c), the difference in the air voltage value between the PDMS device and those made of BIO and HTL resins is reduced by approximately 32% for both of them. Moving to the water voltage values, those detected at a laser power of 1 mW (Figure 11b) for the MoF devices made of BIO and HTL resins are lower than 60% and 61%, respectively, compared to those of the PDMS device. Focusing on the collected voltage values for water detection at 3 mW (Figure 11d), for the BIO and HTL MoF devices, they are lower by 31% and 32%, respectively, than the PDMS one. When the input laser power is set at the highest considered value, i.e., 5 mW (Figure 11e), the difference in the air voltage value between the PDMS device and those made of BIO and HTL resins is reduced by approximately 14% and 13%, respectively.

Moving on to the last operative condition, {Fluid, Input Laser Power} = {water; 5 mW}, the voltage signals are practically equally recognized for the three different selected materials. In fact, the associated curves are almost overlapped, as shown in Figure 11f. This outcome is justified by the 10 V output voltage limit specified in the photodiode TDS. As a result, the saturation value is reached in correspondence with this operative condition (about 11 V), leading to the same water voltage value detected from the three different MoF devices. At this operative condition, it would seem that an optimum operating point has been reached for the two investigated 3D printable resins since it looks like the three devices can discriminate the two investigated fluids similarly. However, the photodiode output voltage limit must be taken into account, which, when the PDMS MoF is used for the detection, takes the detected water voltage signal at the saturation value even when the input laser power is set at 1 mW. Thus, by setting the laser power at 5 mW, it is highly probable that the actual water voltage signal detected from the PDMS device would be higher, providing a higher voltage range value than the resins’ ones. This expected result would be consistent with the PDMS better discrimination capability, supported by its optical properties previously discussed.

The discussed signals were obtained through post-processing of the optical signal acquisition, which was conducted for 30 s, following the procedures outlined in Section 2.4.1. Both the calculated average values for a 30 s acquisition time window and the calculated voltage range (ΔV) are reported in Table 7.

**Figure 12:**
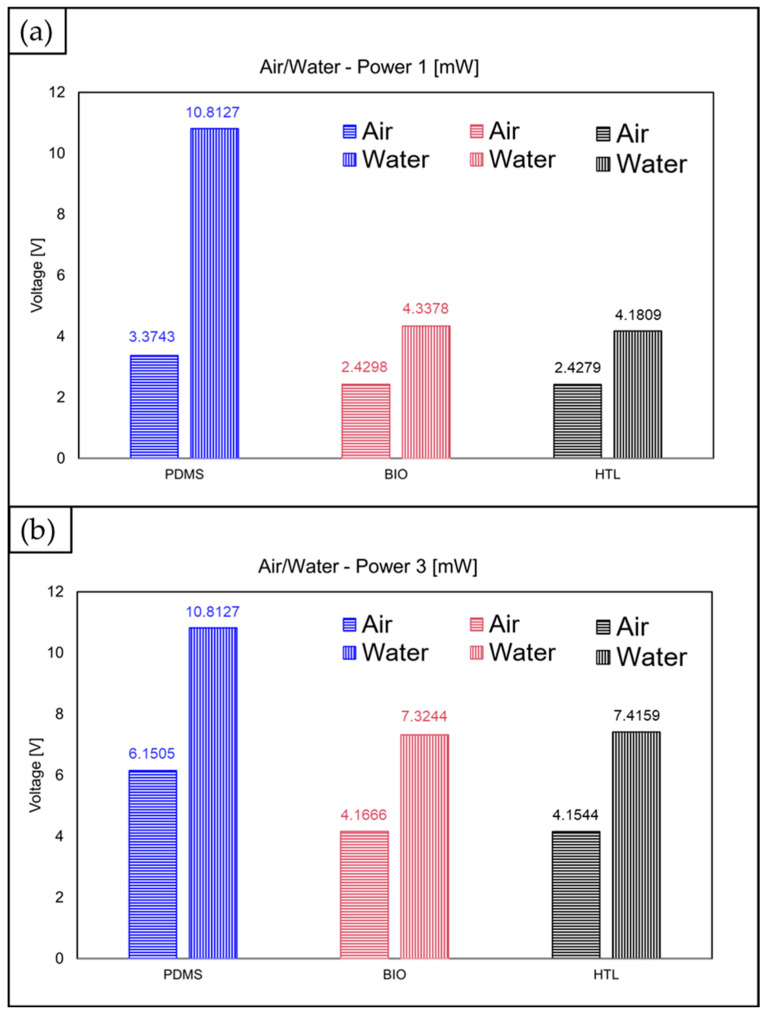
Mean values for each acquired optical signal from the three MoF devices made of PDMS (blue), BIO (red), and HTL (black) resins: detection at a laser power of 1 mW (**a**) and 3 mW (**b**) for either air (horizontal background) or water (vertical background). Bar errors were not added because they were significantly narrower than the bar height.

The mean values of the acquired optical signals, related to the three devices and calculated in correspondence to an input laser power of 1 mW and 3 mW, are reported in Figure 12. In the operative condition with the laser power at 1 mW, the difference between the water and air signals was higher for the PDMS, being ΔV = 7.4 V, compared to the BIO and HTL devices, which have range values equal to ΔV = 1.9 V and ΔV = 1.8 V, respectively. This result shows that the PDMS device better discriminates the two investigated fluids since the gap between the water and the air values is larger. As illustrated in Figure 4, the full transparency of PDMS compared to BIO and HTL’s yellow-like resins justifies this outcome. Raising the laser input power to a value of 3 mW makes the range values comparable for the three devices: ΔV = 3.7 V for PDMS, ΔV = 3.2 V for BIO, and ΔV = 3.3 V for HTL resins. Once again, this result is justified by the fact that, even though the PDMS is projected to have higher voltage values than yellow resins due to its higher transparency (see Figure 4) and lower refractive index value (*n* = 1.4118), it must be taken into account the photodiode output voltage limit. Indeed, saturation for the water voltage signal is already achieved at the lowest value set for the input laser power (1 mW), which does not increase proportionally with the laser power rise. Contrarily, similar voltage values for the BIO and HTL resins, which result in almost totally overlapped curves, are driven by their nearly identical estimated refractive index values (1.6859 for BIO versus 1.7043 for HTL).

## 4. Conclusions

This experimental work proposed a novel method to estimate the refractive index value within an operative range of (*n_min_* = 1.56; *n_max_* = 1.70) of photocurable 3D printable resins characterized by a smooth surface. It relies on developing a quadratic polynomial regression model showing good performance, i.e., *R*^2^ = 80%. Furthermore, to the best of our knowledge, a novel, simple, cost-effective, and efficient experimental setup is presented in this study for the first time.

The method was implemented to determine the refractive index value for two novel 3D printable resins (commercially named BIO and HTL) compatible with the innovative PμSTL 3D printing technique. The same was further used to manufacture MoF devices, which were first optically characterized to assess their suitability to run two-phase flow detection and control. Next, their optical performances were compared to the PDMS one, which was used as the material to realize the same device. Based on the obtained results, it was demonstrated that the investigated materials were suitable for the intended purpose. They were able to discriminate between two different fluids characterized by different refractive index values (air and water). However, it should be noted that their discrimination capability was lower than that of PDMS polymer, which is considered the gold standard polymer in the LOC application fields with a refractive index of *n_PDMS_* = 1.4118. This result is justified because the estimated refractive index values for the BIO and HTL resins (*n_BIO_* = 1.6859 and *n_HTL_* = 1.7043, respectively) are higher than the PDMS one.

The obtained results paved the way for the opportunity to rapidly assess if novel resins developed for VA 3D printing techniques may be suitable for MoF devices where the optical method for non-invasive detection of biological and chemical fluids is exploited. Indeed, the two tested resins (BIO and HTL) were characterized by estimated refractive index values located near the upper limit of the operative range for the refractive index value, corresponding to the worst condition in terms of an optical property. All the novel 3D printable resins that will be developed in the near future can be considered suitable for MoF device manufacturing (relying on the same working principle) if their estimated refractive index value is lower than 1.70.

In this sense, Equation (5) provided in this paper has twofold utility. First, it represents a useful tool for unknown refractive index estimation of solid, transparent samples showing a smooth surface. Second, it provides a quick method to evaluate the suitability of novel developed 3D printable resins for the fabrication of MoF devices.

In the end, the proposed method for transmission value measurements can be used in future works to design and develop MoF devices with integrated surface plasmon resonance (SPR) sensors [66,67]. The perfect combination of refractive index values between the core and cladding elements is of utmost importance in such applications. Indeed, according to the waveguide working principle, to obtain a performing waveguide, the core must have a higher refractive index value than the cladding [24]. This class of MoF devices would represent a breakthrough in diagnostic technology, allowing for simultaneous *specific* and *non-specific* information collection from biological and chemical samples.

## Figures and Tables

**Table 1:** Norland Optical Adhesives (NOA) main properties.

NOA Commercial Name	Refractive Index Value	UV-Curing Energy
NOA88	1.56 @ 589 nm	5 J/cm^2^
NOA160	1.60 @ 589 nm	6 J/cm^2^
NOA1665	1.665 @ 589 nm	6 J/cm^2^
NOA170	1.70 @ 589 nm	6 J/cm^2^

**Table 2:** *Device 1* and *Device 2* roughness measurements [10].

MoF Device	Material	Ra [nm]	RMS [nm]	Peak to Peak [nm]
*Device 1*	PDMS	1.097	0.763	35.158
*Device 2*	HTL resin	47.056	37.324	259.121

**Table 3:** Experimental plan: factors and levels.

Factor	Symbol	Type	Unit	Levels (*a* = 4)			
Material	A	Categorical	[-]	NOA88	NOA160	NOA1665	NOA170

**Table 4:** ANOVA table for the selected quadratic polynomial regression model.

	Source	DF	SS	MS	F	*p*
Regression	Linear	1	379,065	-	154.76	0.000
	Quadratic	1	7815	-	3.37	0.074
	Error	41	95,059	2319		
	Total	43	481,939			
	**S**	48.1508				
	***R*** **^2^**	80.28%				
	***R*** **^2^** **-adj**	79.31%				

**Table 5:** Collected transmission measurements for the photocurable 3D printable BIO and HTL resins for each replication (*n* = 1, 2, 3).

Resin	Replication (*n*)	T [%]
BIO	1	151.564
BIO	2	150.701
BIO	3	151.564
HTL	1	104.113
HTL	2	102.558
HTL	3	103.515

**Table 6:** Estimated refractive index value for BIO and HTL 3D printable resins for each replication (*n* = 1, 2, 3) of the transmission value measurements (*T*). Two roots for the second-order Equation (5) were determined for each measured *T*.

Resin	Replication (*n*)	${\hat{R}}_{1}$	${\hat{R}}_{2}$
BIO	1	1.3108	1.7040
BIO	2	1.3103	1.7046
BIO	3	1.3106	1.7043
HTL	1	1.3291	1.6858
HTL	2	1.3287	1.6861
HTL	3	1.3291	1.6858

**Table 7:** Sample mean value ± st. deviation observations [V] and calculated range (ΔV) [V] for all the investigated scenarios. Factors: Fluids = {Air, Water}; Input Laser Power = {1, 5} mW.

	PDMS			BIO			HTL		
	1 [mW]	3 [mW]	5 [mW]	1 [mW]	3 [mW]	5 [mW]	1 [mW]	3 [mW]	5 [mW]
**Air**	3.3743 ± 1.30 × 10^−3^	6.1505 ± 1.10 × 10^−3^	7.0920 ± 7.91 × 10^−2^	2.4298 ± 1.90 × 10^−4^	4.1666 ± 3.30 × 10^−3^	6.0996 ± 7.02 × 10^−2^	2.4279 ± 5.30 × 10^−2^	4.1544 ± 2.10 × 10^−3^	6.1471 ± 2.5 × 10^−3^
**Water**	10.8127 ± 2.15 × 10^−6^	10.8127 ± 2.15 × 10^−6^	10.8127 ± 2.15 × 10^−6^	4.3378 ± 2.5 × 10^−3^	7.3244 ± 2.8 × 10^−3^	10.8494 ± 8.26 × 10^−4^	4.1809 ± 4.44 × 10^−2^	7.4159 ± 2.4 × 10^−3^	10.8487 ± 6.28 × 10^−4^
**Range**	7.4	3.7	3.8	1.9	3.2	4.7	1.8	3.3	4.7

## Data Availability

Data will be made available on request.
